# The Potential Role of NFAT5 and Osmolarity in Peritoneal Injury

**DOI:** 10.1155/2015/578453

**Published:** 2015-09-30

**Authors:** Harald Seeger, Daniel Kitterer, Joerg Latus, Mark Dominik Alscher, Niko Braun, Stephan Segerer

**Affiliations:** ^1^Division of Nephrology, University Hospital Zurich, 8091 Zurich, Switzerland; ^2^Institute of Physiology and Zurich Center for Integrative Human Physiology (ZIHP), University of Zurich, 8057 Zurich, Switzerland; ^3^Division of Nephrology, Department of Internal Medicine, Robert-Bosch-Hospital, 70376 Stuttgart, Germany

## Abstract

A rise in osmotic concentration (osmolarity) activates the transcription factor Nuclear Factor of Activated T Cells 5 (NFAT5, also known as Tonicity-responsive Enhancer Binding Protein, TonEBP). This is part of a regulatory mechanism of cells adjusting to environments of high osmolarity. Under physiological conditions these are particularly important in the kidney. Activation of NFAT5 results in the modulation of various genes including some which promote inflammation. The osmolarity increases in patients with renal failure. Additionally, in peritoneal dialysis the cells of the peritoneal cavity are repeatedly exposed to a rise and fall in osmotic concentrations. Here we review the current information about NFAT5 activation in uremic patients and patients on peritoneal dialysis. We suggest that high osmolarity promotes injury in the “uremic” milieu, which results in inflammation locally in the peritoneal membrane, but most likely also in the systemic circulation.

## 1. Introduction

During the last decades, we have witnessed a rapid growth of the patient population in need for renal replacement therapy [1]. Peritoneal dialysis is the most common form of home dialysis and favoring peritoneal dialysis (PD) as first strategy termed “PD first” is widely recommended [2]. Yet, due to various factors such as misconceptions concerning contraindications to PD, physician training, and apparent simplicity of HD initiation, but also superior reimbursement for hemodialysis in some places, this policy has hitherto not been consequently implemented [3]. “Uremia” has been described as an inflammatory state, caused by a myriad of factors accumulating in our patients with progressive loss of renal function [4]. In addition to this proinflammatory milieu, in patients on peritoneal dialysis we implant a catheter and expose them to dialysate. This is a lifesaving therapy but exposes the patients to additional “stressors.” These include a foreign body reaction (catheter), glucose toxicity with induction of advanced glycation endproducts and formation of glucose degradation products, mechanical stress, changes in pH, and repeated exposure to a high osmotic concentration. Over time these “stressors” cause the peritoneal membrane to deteriorate, which is a major contributor to treatment failure in patients on PD [5–7]. We became interested in studying the response of peritoneal cells and biopsies from patients on PD concerning the response to osmolarity as one “puzzle stone” which could contribute to the decreased longevity of the peritoneal membrane.

## 2. Current View of the Mechanisms Leading to Peritoneal Fibrosis

The biological membrane used in peritoneal dialysis is a complex network of various cell types (e.g., mesothelial cells, peritoneal fibroblasts, inflammatory cells, vascular endothelial cells, and pericytes) and matrix components. The peritoneal membrane thickens with a progressive accumulation of submesothelial matrix, which already reaches a significant level prior to the start of PD [8]. In this “uremic” phase, factors not related to the PD procedure already pave the way to the so-called simple peritoneal fibrosis. Later the clinical decrease in ultrafiltration capacity is associated with an expansion of the extracellular matrix (peritoneal fibrosis), formation of small vessels (neoangiogenesis), vasculopathy, increased number of lymphatic vessels, and loss of mesothelial cells (denudation) [9–13].

## 3. Cellular Responses to Osmotic “Stress”

The osmosensitive transcription factor NFAT5 plays a key role in the protection of cells against an increase in osmotic concentration [14]. It regulates the expression of genes which in part counteract the high osmolarity. Therefore it induces genes involved in the production and uptake of organic osmolytes. The role of NFAT5 in hyperosmolar conditions has been most intensively studied in kidney cells, as in the renal medulla cells typically face high concentrations of urea and sodium chloride [14]. The cellular response to high extracellular solute concentrations is highly conserved in evolution and not confined to the kidney. Consequently, expression of NFAT5 has been demonstrated to be ubiquitous throughout the entire organism. The prototypical reaction of mammalian cells exposed to high extracellular osmolarity is immediate shrinkage due to water efflux via aquaporins. Subsequently, the cell actively increases the concentration of intracellular organic osmolytes such as taurine, betaine, inositol, sorbitol, and glycerophosphocholine (GPC) via expression of certain enzymes such as aldose reductase (AR) or transporter molecules such as the betaine/GABA transporter (BGT1), the sodium/myoinositol cotransporter (*SLC5A3*), or the taurine transporter (TauT), thus equilibrating intra- and extracellular osmolar pressure. The above enzymes and transporters have been shown to be transcriptionally coregulated by NFAT5 (reviewed in [14]).

Certain chronic inflammatory conditions are associated with local or systemic hyperosmolality. For example, in patients with diabetes mellitus intermittent hyperglycemia is associated with increased plasma concentrations of the proinflammatory cytokines tumor necrosis factor- (TNF-) *α*, Interleukin- (IL-) 6, and IL-18 [15]. In peripheral blood mononuclear cells from diabetic patients with chronic microvascular lesions increased NFAT5 DNA binding activity was demonstrated [16]. IL-1*β*, IL-6, and IL-18 display NFAT5 target sequences in their promoter [15], TNF-*α* and LT*β* are known target genes of NFAT5 in inflammatory cells [17], and exposure of human peripheral blood mononuclear cells to osmotic stress resulted in increased expression of IL-1 and IL-8 [18]. Consequently, in diabetic patients systemic hyperosmolality might lead to NFAT5 mediated release of proinflammatory cytokines.

Osmotic stress also increases nuclear factor-*κ*B (NF-*κ*B) activity, a key regulator in the induction of inflammatory responses via the regulation of chemokines, cytokines, and growth factors. Induction of NF-*κ*B by hyperosmolarity involves p38 kinase and is independent of NFAT5; however, NF-*κ*B induced transcription of target genes is significantly enhanced by binding to NFAT5 [19]. On the other hand, it is plausible that cytokines such as TNF*α* or LT*β*, which are released via hyperosmolarity induced NFAT5 activation [17], lead to secondary activation of the NF-*κ*B pathway. One of the most extensively studied chemokines is chemokine (C-C motif) ligand 2 (CCL2), also known as Monocyte Chemoattractant Protein-1 (MCP-1). It regulates the migration and infiltration of monocytes/macrophages. It can be produced by a variety of cell types after induction by oxidative stress, cytokines, growth factors, and hyperosmolarity [20–23]. In summary there are close interactions between the response to increased osmotic concentrations and the inflammatory cascade.

## 4. Osmotic Concentration in Patients with Renal Failure and on Dialysis

We became interested in studying NFAT5 in PD patients, as with the PD solution the peritoneal cavity is continuously exposed to cycles of a rapid rise and slow fall of osmolarity. An unexpected finding in our studies was a prominent induction of NFAT5 in uremic patients [24].

Other authors demonstrated that osmolarity increases in patients while progressing through the stages of chronic renal failure [25]. The serum osmolarity averaged 294 mosmol/kg H_2_O in patients with normal kidney function and increased to a mean of 323 mosmol/kg  H_2_O in predialytic patients (CKD stage 5). Similar predialysis osmolalities were measured in dialysis dependent patients [25]. These results were confirmed in pediatric patients [26]. The underlying mechanisms are not completely understood, as blood urea does not fully account for the increase in osmolality. A hypothesis is that the retention of unmeasured solutes accounts for the increased osmolality as demonstrated by a rise in the osmolal gap. After a hemodialysis session, the serum osmolality was found to be reduced, but standard hemodialysis is not sufficient to return serum osmolality and osmolal gap back to normal [26, 27]. The chronic hyperosmolar state might be looked at as an “uremic toxin.” Exposure of cells to hyperosmolar stress might result in cell cycle arrest, apoptosis, DNA damage, inhibition of transcription, and translation and might therefore have various detrimental effects [14].

## 5. Osmotic Response of the Peritoneal Membrane

Osmotic pressure has a significant impact on peritoneal cells, which was demonstrated by Breborowicz and others, who showed that exposure of mesothelial cells to hyperosmolar solutions in vitro leads to acute cell shrinkage [28]. Early studies on immortalized and primary human peritoneal mesothelial cells demonstrated that upon incubation with hyperosmolar solutions (osmolytes: glucose, mannitol, or NaCl) the matrix metalloproteinase 9 (MMP9) was downregulated favoring accumulation of collagen type IV, thereby potentially promoting the development of peritoneal fibrosis [29]. The exposure of the peritoneum to hyperosmolar solutions led to vasodilation in another study [30]. This effect was hypothesized to be caused by water efflux through aquaporin-1 channels of the microvascular endothelium, which consecutively leads to NO release by the endothelial cells [31, 32]. Furthermore, induction of apoptosis has been demonstrated by high osmolarity in mesothelial cells [33].

Lee and others were the first to demonstrate that glucose induced the expression of the proinflammatory cytokine CCL2 in human peritoneal mesothelial cells, thereby potentially favoring the influx of proinflammatory cells [21]. The effects were mediated by glucose itself, since mannitol did not stimulate CCL2 release. In contrast Matsuo and coworkers used rat peritoneal mesothelial cells (RPMCs) which responded to hyperosmolar concentrations of glucose (2.5% (140 mM) glucose) with increased transcription and release of CCL2. The effect was not glucose specific since it was also observed in cells exposed to the same concentration of mannitol. This implies that the response was elicited by hyperosmolarity and not glucose per se. The release of CCL2 was time and concentration dependent. The effects of hyperosmolarity (glucose or mannitol) on CCL2 mRNA expression were mediated by protein kinase C (PKC), in part via NF-*κ*B activation. It could be blocked by micromolar concentrations of the glucocorticoid prednisolone via inhibition of NF-*κ*B activation mediated by increased expression of I-*κ*B-*α* [22]. The finding that NF-*κ*B activation was only partially responsible for mediating the downstream effects of PKC was consistent with earlier findings that also the tyrosine kinase AP-1 pathway is involved in CCL2 induction elicited by high glucose concentrations in mesothelial cells [21]. The finding that the upregulation of CCL2 is mediated by hyperosmolarity rather than glucose was supported by Wong and others who demonstrated that mannitol had similar effects on human peritoneal mesothelial cells compared to glucose with respect to CCL2 induction [23]. These findings were recently recapitulated in immortalized human mesothelial cells stimulated by hyperosmolar concentrations of glucose, mannitol, or NaCl [34].

Little or no information is currently available on the role of peritoneal fibroblasts or the potential cross-talk between mesothelial cells and mesothelial cells in this complex system. In summary hyperosmolarity can produce a proinflammatory milieu in mesothelial cells.

## 6. Potential Role of the Transcription Factor NFAT5 in Response to Osmotic Stress in Peritoneal Cells

As described above, peritoneal cells are chronically exposed to high osmolality during peritoneal dialysis due to the supraphysiologic osmolality of the dialysis fluids ranging from 380 to 510 mosmol/kg H_2_O, but also to increased serum osmolality in the predialytic phase in chronic kidney disease.

**Figure 1:**
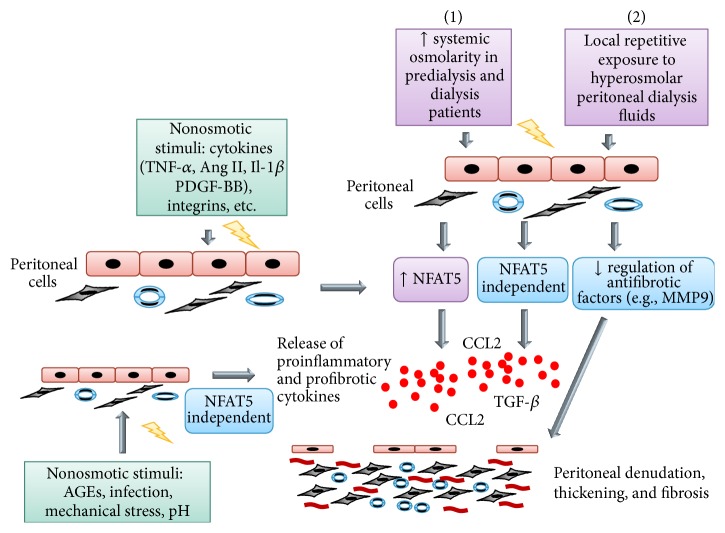
Involvement of osmotic and nonosmotic mechanism in peritoneal fibrosis. (1) Rise in osmolarity stimulates NFAT5 in patients with “uremia.” Activation of NFAT5 results in a counterregulatory response protecting cells from hyperosmolarity but also induces inflammatory mediators (e.g., the chemokine CCL2) and growth factors. Additionally antifibrotic factors are downregulated. (2) Exposure to hyperosmolar dialysate might perpetuate NFAT5 induction. The balance between pro- and antifibrotic factors is shifted towards matrix deposition. Note that there are osmotic and nonosmotic factors inducing NFAT5 and also direct inducers of CCL2, which increases the complexity of the system and might lead to several vicious cycles promoting injury. Abbreviations: TNF-*α* = tumor necrosis factor *α*, Ang II = angiotensin II, PDGF-BB = platelet derived growth factor BB, TGF-*β* = transforming growth factor b, CCL2 = chemokine (C-C motif) ligand 2, MMP9 = matrix metalloproteinase 9, AGE = advanced glycation end products, NFAT5 = nuclear factor of activated T cells 5, and Il-1*β* = interleukin-1*β*.

It has been demonstrated that NFAT5 activation in a hyperosmotic environment leads to the release of proinflammatory mediators [15, 35]. CCL2, especially, was upregulated in renal tubular epithelial cells upon osmotic stress in an NFAT5 dependent manner [19, 36]. This has led several groups to investigate the role of NFAT5 in the response of peritoneal cells to hyperosmolarity. Küper and colleagues demonstrated that NFAT5 activity was increased in human immortalized mesothelial (Met5A) cells upon exposure to hyperosmolar concentrations of NaCl, mannitol, or glucose in an osmolality dependent manner [34]. Peak activation of NFAT5 was reached at 400 mosmol/kg H_2_O for glucose and mannitol and 450 mosmol/kg H_2_O for NaCl and decreased at an osmolality >450 mosmol/kg H_2_O. Interestingly, in renal cells, maximal NFAT5 activation was observed at osmolalities of >500 mosmol/kg H_2_O. Knockdown of NFAT5 by siRNA abrogated CCL2 mRNA and protein upregulation of these cells stimulated by hyperosmolarity [34]. These results point to a crucial role of NFAT5 in CCL2 upregulation by mesothelial cells exposed to osmotic stress. In a further set of experiments the authors uncovered that the transcription factor NF-*κ*B was activated under hyperosmotic conditions and that pharmacologic blockade of NF-*κ*B abolished CCL2 expression. This confirms that NFAT5 and NF-*κ*B cooperate also in cells of mesothelial origin in the stimulation of cytokine release upon osmotic stress as has previously been shown for renal cells ([34], Figure 1). One caveat of this study is that an immortalized cell line (Met5A) was used and results were not confirmed in primary human mesothelial cells.

Human peritoneal fibroblasts (HPFBs) have a crucial role in the pathogenesis of peritoneal fibrosis via release of proinflammatory cytokines and synthesis of extracellular matrix [37]. Our group has thus investigated the response of HPFBs to hyperosmolar glucose solutions in vitro. Upon exposure to increasing glucose concentrations in vitro, NFAT5 mRNA was significantly upregulated in an osmolality dependent fashion in these cells after six hours. Yet, after 24 and 96 hours no further increase of NFAT5 mRNA could be observed compared to controls. CCL2 mRNA was only induced after six hours in the cells exposed to the highest glucose concentration (125 mosmol/kg H_2_O). However, after 96 hours a robust, concentration dependent upregulation of CCL2 mRNA could be witnessed. Interestingly, CCL2 upregulation occurred at a time point where NFAT5 mRNA levels were not upregulated any longer compared to controls. Therefore, there was a significant delay between NFAT5 mRNA induction and upregulation of CCL2, which could be explained by two scenarios. First, the delay between NFAT5 mRNA induction and upregulation of CCL2 might be explained by the time necessary for the translation of NFAT5 mRNA, synthesis of the protein, and nuclear translocation of the transcription factor before downstream targets can be induced. Secondly the more likely scenario is that CCL2 could have been induced in an NFAT5 independent fashion [24].

In further experiments, peritoneal biopsies were evaluated from uremic patients before the start of peritoneal dialysis and patients already on peritoneal dialysis and compared to controls. It was observed that in patients not on PD there was already a thickening of the peritoneal membrane. This has previously been described by other groups [8, 9]. Unexpectedly, NFAT5 expression was increased not only in patients on PD, but also in uremic patients not yet on PD. In healthy controls NFAT5 expression was significantly lower than in the other two groups. There was no significant difference in the expression level between uremic patients and patients on PD. This was true, even when the PD group was split up into patients with <8 h since the last exposure to dialysate and those with >8 h. The stimulus for NFAT5 upregulation in peritoneum of patients prior to the start of PD is unclear. One possibility is that the increase in plasma osmolality in patients with preterminal CKD already suffices to induce NFAT5. Alternatively induction of NFAT5 might have been induced in a non-osmolality-dependent fashion. In fact, various circumstances have been described in which NFAT5 appears to be activated non-osmolality-dependent, for example, during embryonic development and cancer metastasis [38, 39]. Also cross-linking of the T cell receptor in T cells within an isotonic environment resulted in NFAT5 upregulation via calcineurin signaling [40]. In rheumatoid arthritis NFAT5 expression was shown to be increased in the synovium and in synovial fibroblast-like cells the cytokines Il-1*β* and TNF-*α* strongly induced NFAT5 expression [41]. Finally, Haltermann and others recently demonstrated in vascular smooth muscle cells (SMCs) that angiotensin II leads to nuclear translocation of NFAT5 and downstream gene transcription. PDGF-BB resulted in increased NFAT5 protein expression and SMC proliferation and migration [42].

However, many of the above findings face a similar problem, that, particularly in the in vivo studies, it cannot be excluded that a hyperosmolar microenvironment contributed to NFAT5 activation.

Contrary to NFAT5, CCL2 demonstrated a strong induction in the peritoneum of patients on PD, whereas the CCL2 message was only slightly upregulated in patients prior to PD. This was confirmed by immunohistochemistry. In the group prior to PD only occasional staining in MCs was observed; yet the MCs and fibroblasts (FBCs) of patients on PD displayed strong CCL2 immunoreactivity. Finally, while in patients on PD the peritoneal immunostaining for the NF-*κ*B subunits p50 and p65 was markedly positive mainly in FBCs, there was barely immunopositivity in peritoneal FBCs in predialysis patients and no staining in controls [37].

Taken together, the above data strongly suggests that osmotic stress during peritoneal dialysis significantly affects the peritoneum and may contribute to peritoneal inflammation and fibrosis via stimulation of cytokine release from peritoneal cells. Our data suggests that osmotic stress to the peritoneal membrane exists even prior to the commencement of peritoneal dialysis and exposure to hyperosmolar dialysis fluids in uremic patients as evidenced by upregulation of NFAT5 mRNA and protein in peritoneal membranes of predialyis patients. This pathomechanism is fairly conceivable since uremic individuals display significantly increased serum osmolality as described above. Though speculative, it is imaginable that serum hyperosmolality in uremic patients leads to NFAT5 activation within cells of the peritoneal membrane and the release of proinflammatory and profibrotic factors finally leading to thickening of the peritoneal membrane which has been repeatedly observed in predialytic patients.

## 7. Conclusion

Increased osmolarity is the basis of water elimination in PD. There is now evidence in uremic patients and PD patients of an intraperitoneal induction of NFAT5. Osmotic and nonosmotic trigger might be present in PD, which could promote an inflammatory response. Also in mesothelial cells and peritoneal fibroblasts a response to hyperosmolar dialysate has been illustrated; until now the role of NFAT5 induced by dialysate needs to be shown in vivo. The blockade of NFAT5 might inhibit the inflammatory response and needs to be studied in models of peritoneal injury.
